# Carbon Dots-Mediated Photodynamic Treatment Reduces Postharvest Senescence and Decay of Grapes by Regulating the Antioxidant System

**DOI:** 10.3390/foods13172717

**Published:** 2024-08-27

**Authors:** Zhi-Jing Ni, Ying Xue, Wei Wang, Juan Du, Kiran Thakur, Wen-Ping Ma, Zhao-Jun Wei

**Affiliations:** 1 School of Biological Science and Engineering, North Minzu University, Yinchuan 750021, China; lovebear@vip.163.com (Z.-J.N.); xueying15132907710@163.com (Y.X.); wwang@nun.edu.cn (W.W.); 20217528@stu.nun.edu.cn (J.D.); kumarikiran@hfut.edu.cn (K.T.); 2008025@nun.edu.cn (W.-P.M.); 2 School of Food and Biological Engineering, Hefei University of Technology, Hefei 230009, China

**Keywords:** grapes, carbon dots-mediated photodynamic treatment, antioxidant enzymes, postharvest senescence

## Abstract

Grapes are susceptible to mold and decay during postharvest storage, and developing new technologies to extend their storage period has important application value. Photodynamic technology (PDT) in concurrence with carbon dots (CDs) proposes an innovative and eco-friendly preservation strategy. We examined the effects of carbon dots combined with photodynamic treatment on postharvest senescence and antioxidant system of table grape. The compounding of photodynamic technology with a 0.06 g L^−1^ CDs solution could possibly extend the postharvest storage period of grape berries. Through this strategy, we achieved a decreased rate of fruit rotting and weight loss alongside the delayed deterioration of fruit firmness, soluble solids, and titratable acid. As paired with photodynamic technology, CDs considerably decreased the postharvest storage loss of phenols, flavonoids, and reducing sugars as compared to the control group. Concurrently, it remarkably postponed the build-up of hydrogen peroxide (H_2_O_2_), superoxide anion (O_2_^∙−^), and malondialdehyde (MDA); elevated the levels of reduced ascorbic acid (AsA) and reduced glutathione (GSH); lowered the levels of dehydroascorbic acid (DHA) and oxidized glutathione (GSSG); raised the ratios of AsA/DHA and GSSH/GSSG; encouraged the activities of superoxide dismutase (SOD) and phenylalanine ammonia-lyase (PAL); and inhibited the activities of polyphenol oxidase (PPO) and lipoxygenase (LOX). Furthermore, it enhanced the iron reduction antioxidant capacity (FRAP) and DPPH radical scavenging capacity of grape berries. CDs combined with photodynamic treatment could efficiently lessen postharvest senescence and decay of grape berry while extending the storage time.

## 1. Introduction

The grape *Vitis vinifera* × *V. labrusca* ‘Kyoho’ is unique to Japan and is one of the main table grape varieties. It is large, plump, sweet-flavored, and enriched in nutrients, minerals, polyphenols, and flavonoids, among others; it is also resilient to oxidative stress and exhibits anti-aging properties [1]. Grapes are susceptible to mold and decay during postharvest storage. Conventional grape preservation techniques primarily concentrate on physical and chemical preservation as well as novel preservatives like ultraviolet irradiation [2], sulfur dioxide preservatives [3], hydrogen sulfide fumigation [4], 1-MCP treatment [5], and essential oil packaging materials [6]. The key to deepening the research on table grapes is to discover novel, ecologically friendly preservation technology because many of the current methods pose risks to the fruit’s flavor, leave chemical residues in the environment, contaminate the ecosystem, and cause other problems [5].

Recent research highlights the application of photodynamic technology (PDT) in food preservation [7]. Its underlying principle is based on the excited photosensitizer at certain wavelengths of light that is responsible for production of reactive oxygen species molecules and inactivation of microbial cells through oxidation [8]. Photodynamic reaction comprises two different reaction types taking place simultaneously. Type I can produce reactive oxygen species that cause damage to proteins and other macromolecules of microbial cells and then inactivate target cells [9]. Despite their poorly water solubility and chemically unstable nature [10], some natural photosensitizers, namely curcumin [11], chlorophyll [12], riboflavin [13], coumarin [14], and other naturally occurring photosensitizers, etc., have become potential photosensitizers for food preservation. To address the existing constraints, the development of a novel photosensitizer is imperative [8].

Carbon dots (CDs) are novel materials with intriguing characteristics that have garnered significant attention due to their desirable properties, including water solubility, low toxicity, antimicrobial activity, and ease of synthesis and modification [15]. The presence of hydrophilic functional groups (C=C and distinct elements) on the edge or underlying plane of carbon dots contributes to their excellent water solubility. Studies have demonstrated that CDs exhibit good biocompatibility, posing minimal harm to organisms and being excretable through normal physiological metabolism, as evidenced by experiments involving zebrafish [10]. In terms of their antimicrobial role, modified CDs were found to kill almost all the bacteria on the surface of the film in the presence of blue light at 470 nm for 60 min [16]. Jhonsi et al. [17] found that CDs also have a protective effect against *Candida albicans*.

Various synthesis methods have been developed for CDs, including the “top-down” method [18] utilizing graphite and activated carbon as well as the “bottom-up” synthesis method [19] employing different carbon sources such as sugars and organic salts. Among them, the “bottom-up” synthesis method is particularly notable for its speed, simplicity, cost effectiveness, and biocompatibility, making it a focus of interest across diverse fields [20]. Recent research emphasizes new synthesis methods for CDs and their potential in food antibacterial preservation and safety testing. For example, banana-derived CDs showed noticeable antibacterial effects, which could prolong the storage period of soybean milk by 1–3 days [21]. On the other hand, fluorescent probe broccoli-derived CDs were found to detect food/water contamination [22]. Overall, CDs’ distinct qualities and wide range of uses make them an intriguing subject for study, with prospective applications in a variety of industries, such as environmental monitoring, food science, and medicine.

One previous study conducted hydrothermal synthesis using citric acid and ethylenediamine to produce nomenclature, a method known for its simplicity, cost effectiveness, short synthesis time, and high yield [23]. Du et al. [24] showed that this previously reported CDs-mediated photodynamic technology can extend the storage period of goji berries by 9 days. The use of CDs combined with photodynamic treatment in our research group showed some theoretical basis for the preservation of wolfberry [24]; however, the effect of this treatment method on the preservation of table grapes after harvest was not reported. In order to further extend the use of this preservation strategy in table grape, we used CDs as photosensitizers of photodynamic technology for postharvest preservation of Kyoho grape. The effects of photosensitizers concentration and light duration on the storage quality of Kyoho grape were investigated at a light irradiance of 125 mW cm^−2^. Our study can provide a novel approach for the application of CDs-mediated photodynamic technology in postharvest preservation of table grapes. In this study, we mainly focus on how CDs-mediated treatment can reduce postharvest senescence and decay of grapes by regulating the antioxidant system.

## 2. Materials and Methods

### 2.1. Synthesis of Nomenclature

Introduction of carbon dots (CDs): A detailed synthesis and characterization of CDs was described in our previous study [24]. CDs represent non-toxic nanomaterials (4.66 nm) at a concentration of 0.1 g L^−1^.

### 2.2. Experimental Design and Sample Treatment

**Figure 1:**
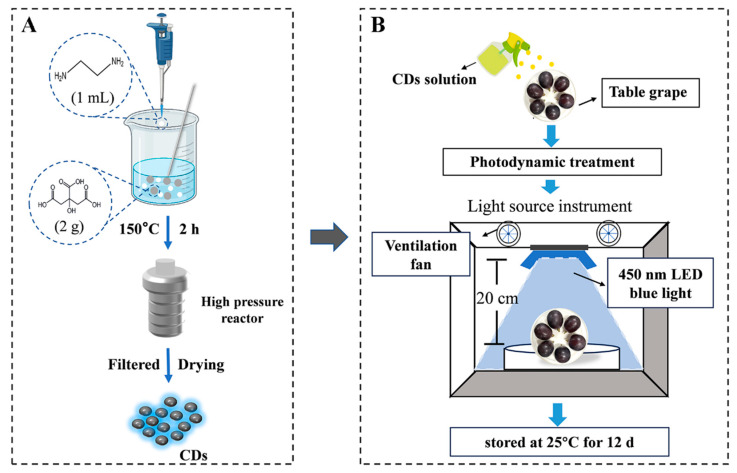
Schematic diagram of CDs-mediated PDT-treated grapes. (**A**) Composite diagram of CDs and (**B**) flow diagram of grape treatment under light.

Following the description by Du et al. [24], blue light (450 nm) was used as the light source, and the blue light irradiance was fixed at 125 mW/cm^2^. Further, with the optimal carbon dot concentration of 0.06 g L^−1^ and the light time of 5 min for treatment, we designed four experimental groups: the optimal treatment group (P+L+), the control group (P-L-), the group with photosensitizer and no light (P+L-), and the group with light without photosensitizer (P-L+). We verified each group by appearance and decay rate (Figure 1). L: illumination; P: photosensitizer (carbon dots); +: light or add; -: not illuminated or added.

The table grapes (1300) were procured from the wholesale fruit market in Xixia District, Ningxia, and the test variety was Kyoho grape (*V. vinifera* × *V. labrusca* ‘Kyoho’). Freshly purchased grapes of the same size and ripeness and without physical damage were selected. After disinfecting with 0.1% sodium hypochlorite solution, grapes were washed with distilled water and naturally dried for later use. CDs solution with a concentration of 0.06 g L^−1^ was evenly sprayed on the surface of fresh grapes. The sprayed grapes were placed in a row on a work table 20 cm away from the blue light source and irradiated for 5 min, and the reverse operation was performed to ensure the uniform light exposure. Blue light (450 nm) was used as the light source, and the blue light irradiance was fixed at 125 mW cm^−2^ (Figure 1B), and the blank group was sprayed with distilled water. The treated fruits were placed in a freshness preservation box (13.2 cm × 8.2 cm × 6 cm), and each box with 16 grapes was stored at room temperature, away from light (25 °C humidity 38%) conditions (Supplemental Figure S1).

### 2.3. Evaluation of Decay Rate, Weight Loss, and Hardness of Grapes

The rotting rate and weight loss rate of the grapes were measured every two days (16 grapes were selected for each repeat, and the experiment was repeated three times); in addition, the skin and pulp of the sample (30 grapes, a total of three replicates) were separated, frozen with liquid nitrogen, and stored in an ultra-low temperature refrigerator of −80 °C for the determination of other indicators. In accordance with the assay method of Lv et al. [25], the fruit rot rate, weight loss rate, and hardness were determined every two days, and the hardness was determined according the following formula:

(1) $$\mathrm{Fruit}\;\mathrm{decay}\;\mathrm{rate}=\frac{\mathrm{number}\;\mathrm{of}\;\mathrm{rotten}\;\mathrm{fruits}}{\mathrm{total}\;\mathrm{number}\;\mathrm{of}\;\mathrm{fruits}}\times 100\%$$

(2) $$\mathrm{Weight}\;\mathrm{loss}\;\mathrm{rate}=\frac{(W1-W2)}{W1}\times 100\%$$

where W1 is the quality of grapes on the 0 day of storage, and W2 is the mass of grapes every two days during storage. The result was measured in g.

Grape hardness was determined using a texture analyzer (TMS-PRO, FTC, New York, NY, USA), and a cylindrical probe with a diameter of 0.2 cm was used to measure hardness in the equatorial part of the grape. The maximum force was set to 100 N, the test speed was 30 mm/min, the deformation was 30%, the penetration distance was 5 mm, and the trigger force was 0.15 N. The average of the 12 grapes tested was taken, and the experiment was repeated three times. The results are expressed in Newton (N).

### 2.4. Evaluation of Total Soluble Solids and Titratable Acids 

According to the assay method of Elam et al. [26], the total soluble solids (TSS) and titratable acids (TA) of grapes were determined using a glucose–acid analyzer. After peeling the grapes, the juice was squeezed out with four layers of gauze, and the TSS of the grapes was determined by sucking the grape juice and dropping it in the groove of the sample. The TA of the grapes was determined by the tester, the unit is presented as %, and the total soluble solids/titratable acids (TSS/TA) ratio was calculated. Using a set of 12 grapes, the experiment was repeated three times

### 2.5. Evaluation of Superoxide Anion, Hydrogen Peroxide, and Malondialdehyde Contents

The contents of superoxide anion (O_2_^∙−^), hydrogen peroxide (H_2_O_2_), and malondialdehyde (MDA) were determined using a kit (Suzhou Comin Biotechnology Co., Ltd., Suzhou, China). For the determination of O_2_^∙−^ content, 0.1 g of grape skin and 1 g of grape pulp were added to the extraction solution and transferred to the ice bath and centrifuged at 10,000× *g* for 20 min at 4 °C. The supernatant was allowed to react with hydroxylamine hydrochloride to form a red azo compound, and there was a special absorption peak at 530 nm. For H_2_O_2_ and MDA determination, 0.1 g of grape skin and 2 g of pulp were added to the extraction solution, ground in an ice bath, and centrifuged at 8000× *g* for 10 min at 4 °C, and the supernatant was taken for testing. The determination of H_2_O_2_ content was confirmed with the fact that hydrogen peroxide and titanium sulfate produce a yellow titanium peroxide complex with special absorption at 415 nm. The content of MDA was determined by determination of thiobarbituric acid, and the product formed by MDA showed a maximum absorption peak at 532 nm. The content of O_2_^∙−^ was expressed by nmol g^−1^ FW, and the contents of H_2_O_2_ and MDA were expressed by nmol g^−1^ FW. All the above-mentioned treatments were repeated three times.

### 2.6. Evaluation of Total Phenols, Flavonoids, and Reducing Sugars Contents

The contents of total phenols, flavonoids, and reducing sugars in grape pulp and skin were determined by kit (Suzhou Comin Biotechnology Co., Ltd., Suzhou, China). For total phenols and flavonoids, 5 g samples were dried to constant weight and crushed through 40 mesh sieves. Then, 0.06 g of skin and pulp were weighed, 2 mL of 60% ethanol was added for extraction, and after two hours of oscillating extraction at 60 °C, the oscillating extracted samples were centrifuged at 10,000× *g*, 25 °C for 10 min to obtain the supernatant for testing. Tungsten molybdate can be reduced by phenols, and the total phenol content of the sample was determined at 760 nm. In alkaline nitrite solution, flavonoids can react with aluminum ions in a yellow complex with a special peak at 510 nm, and the absorbance value at 510 nm can be determined to calculate the content of flavonoids. The change in absorbance at 540 nm indicated the reducing sugar content. All the assay results are expressed as g kg^−1^ FW.

### 2.7. Determination of Antioxidant Enzyme Activity and Ascorbic Acid–Glutathione Circulating System Substances

#### 2.7.1. Antioxidant Enzyme Activity Evaluation

According to Lv et al. [25], the activities of superoxide dismutase (SOD), phenylalanine ammonia-lyase (PAL), polyphenol oxidase (PPO), and lipoxygenase (LOX) were determined using the kit procured from Suzhou Comin Biotechnology Co., Ltd. For the determination of superoxide dismutase and polyphenol oxidase, the specific operation steps were as follows: 0.1 g of grape skin and 2 g of pulp were added with the extract for ice bath grinding, followed by centrifugation at 4 °C, and the supernatant was taken out for testing. For the determination of PAL, 0.1 g of grape skin and pulp was weighed, and the extract provided by the kit was added, followed by grinding in an ice bath, centrifugation (10,000× *g*) at 4 °C, and removal of the supernatant for testing. For the determination of LOX, 0.1 g of grape skin and 2 g of pulp were weighed, the extracts provided were added and ground in an ice bath and centrifuged (16,000× *g*) at 4 °C, and the supernatant was taken for testing. All the enzyme activity is expressed in U/g.

#### 2.7.2. Determination of Reduced Ascorbic Acid, Reduced Glutathione, Dehydroascorbic Acid, and Oxidized Glutathione

In accordance with the description of Zhang et al. [3], the contents of ascorbic acid (AsA) and glutathione (GSH), dehydroascorbic acid (DHA), and oxidized glutathione (GSSG) in the ascorbic acid–glutathione (AsA-GSH) circulation system of grape skin and pulp were determined; the kit protocol (Suzhou Comin Biotechnology Co., Ltd., Suzhou, China) was slightly modified, and the experimental procedure was performed in line with the kit instructions. The AsA content is expressed as g10^3^ kg^−1^, the DHA content is expressed as nmol g^−1^, and the GSH and GSSG content is expressed as mmol kg^−1^.

### 2.8. Determination of Total Antioxidant Capacity

Following the description by Xiong et al. [27], the total antioxidant capacity index was measured by a kit (Suzhou Comin Biotechnology Co., Ltd., Suzhou, China), and the DPPH radical scavenging capacity and iron reduction antioxidant level (FRAP) were measured, and the results were calculated by the absorbance values at 515 nm and 593 nm. Their unit is mmol/kg Trolox FW, and all the experiments were performed with three independent replicates.

### 2.9. Statistical Analysis

Each value was expressed as the average of triplicate replicates, and the experimental data and different analysis were analyzed for variance (ANOVA) using SPSS Statistics software (Version 15.0) with *p* < 0.05 level, and graphs were plotted using Origin 2023 software.

## 3. Results and Discussion

### 3.1. Effect of Different Treatments on the Apparent and Decay Rate of Postharvest Grapes

**Figure 2:**
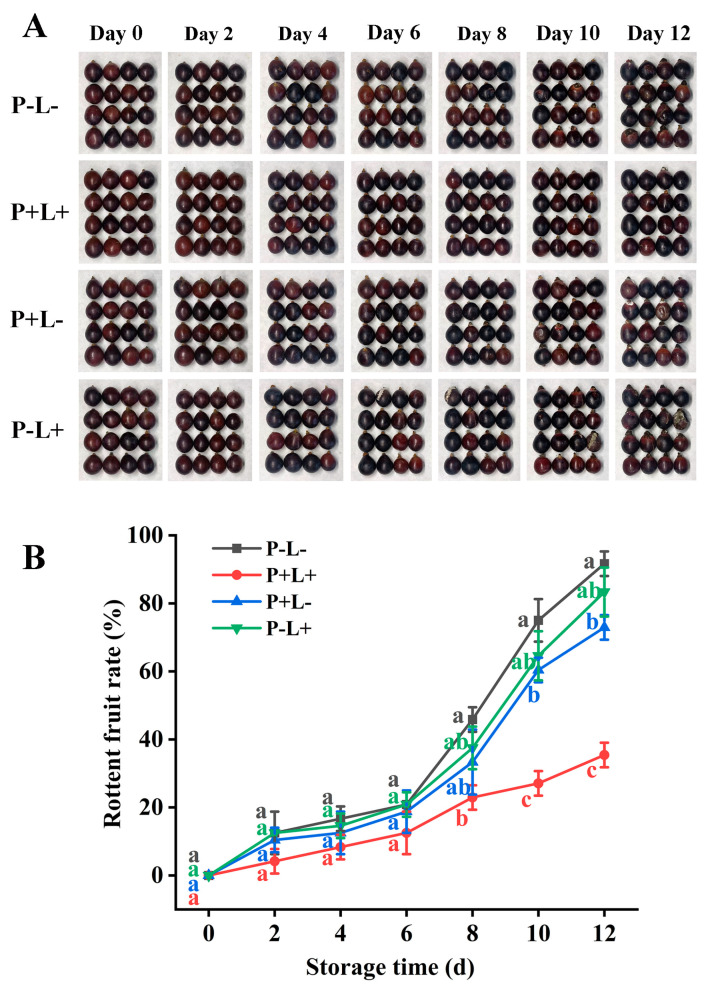
Effects of different treatment conditions on the overall grape appearance (**A**) and decay rate (**B**). P-L-: control group; P+L+: spray 0.06 g L^−1^ CDs solution and 450 nm LED light for 5 min; P+L-: CDs treatment separately (0.06 g L^−1^) without illumination; P-L+: 450 nm LED alone for 5 min, no CDs treatment. After different treatments, the Kyoho grapes were stored at 25 °C for 12 days. The data are expressed as the mean ± SD (standard deviation) (n = 3). Different letters indicate significant difference between treatments (*p* < 0.05).

According to the results of previous experiments [24], the CDs concentration was selected as 0.06 g L^−1^, and the illumination time was set at 5 min for the follow-up experiment. From the grape appearance following the validation experiment (Figure 2A), it can be seen that with the extension of storage time, the decay degree of grape fruit in the other experimental group was more obvious than that in the P+L+ group. On the 12th day of storage, the decay rates of the P-L- group, P+L- group, and P-L+ group were 56.25%, 37.5%, and 47.92% higher than those of the P+L+ group, respectively (Figure 2). From this result, it was evident that CDs combined with photodynamic treatment was the key to fruit preservation rather than photosensitizers or irradiation itself. It is convincing that the stimulation of CDs by blue light at 450 nm to produce singlet oxygen inhibited the microbial activity on the fruit surface and achieved the freshness effect on grapes, which is consistent with the study of Niu et al. [28]. Therefore, 125 mW cm^−2^–0.06 g L^−1^-5 min was used for follow-up experiments throughout the study.

### 3.2. Effects of Carbon Dots Combined with Photodynamic Treatment on Decay Rate, Weight Loss Rate, and Hardness of Grape Berry during Storage

**Figure 3:**
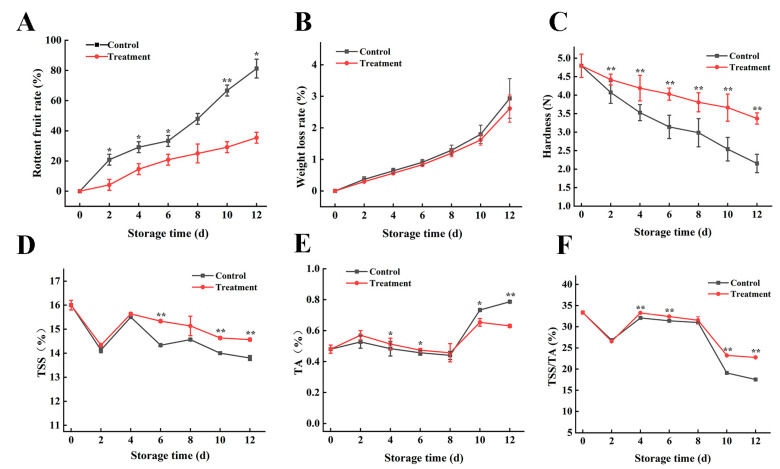
Effect of CDs-mediated photodynamic on the physical properties of postharvest grapes. The rotten fruit rate of fresh grape during storage period; weight loss rate; firmness; and TSS content, TA content, and TSS/TA (**A**–**F**). Treatment group: 0.06 g L^−1^ CDs solution and blue light irradiation for 5 min; control group: distilled water instead of treatment and storage at 25 °C for 12 days. * and ** represent significant differences between the sample point treatment and control groups, where * represents *p* < 0.05, and ** represents *p* < 0.01, and data are presented as mean ± SD (standard deviation) (n = 3).

In postharvest fruits, the decay rate (Figure 3A) and weight loss rate (Figure 3B) of grape berry increased in a linear trend with the length of storage due to the loss of nutrients [29]. Grape firmness (Figure 3C) showed a downward trend compared with the control group, which was 3.36 N on the 12th day of storage, 1.56 times that of the control group.

### 3.3. Effects on Total Soluble Solids and Titratable Acids Contents

In the two groups, the effects of CDs combined with photodynamic treatment on TSS (Figure 3D), TA (Figure 3E), and TSS/TA (Figure 3F) were significant (*p* < 0.05), and the content was higher than that of the control group, indicating that photodynamic treatment could delay the degradation of TSS and TA in grape berry, maintaining the sugar/acid ratio of grapes as well as the nutritional value of grapes. Studies have shown that water loss through transpiration and degradation of cell wall polysaccharides [30] lead to softening, decay, and loss of nutrients in fruit quality, which may be the reason for the reduction in TSS and TA.

### 3.4. Effect on Superoxide Anion, Hydrogen Peroxide, and Malondialdehyde Contents

The generation of reactive oxygen species is a consequence of cellular metabolism, and the intracellular ROS level is tightly regulated under normal physiological conditions and is in a state of dynamic equilibrium. When cells are subjected to external stress, excessive reactive oxygen species (O_2_^∙−^, H_2_O_2_) can cause membrane lipid damage to cells [31], resulting in an increase in the content of MDA, which is a membrane lipid peroxide produced after cell membrane damage, and its accumulation in large quantities is not conducive to fruit storage. In both groups, the control group accumulated more O_2_^∙−^, H_2_O_2_, and MDA content than the treated group regardless of grape skin or grape pulp.

**Figure 4:**
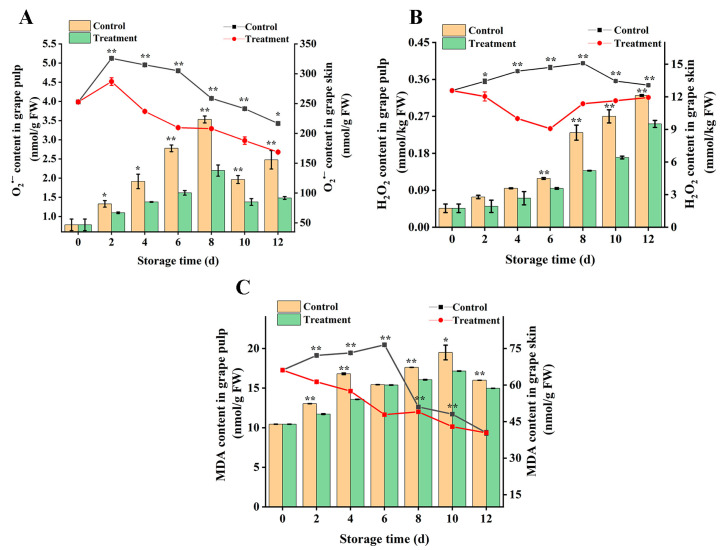
Effect of CDs-mediated PDT of O_2_^∙−^content (**A**), H_2_O_2_ content (**B**), and MDA content (**C**) in grape skin and pulp. Treatment group: 0.06 g L^−1^ CDs solution and blue light irradiation for 5 min; control group: distilled water instead of treatment and storage at 25 °C for 12 days. * and ** represent significant differences between the sample point treatment and control groups, where * represents *p* < 0.05, and ** represents *p* < 0.01, and data are presented as mean ± SD (standard deviation) (n = 3).

The O_2_^∙−^ content of grape berry (Figure 4A) increased first and then decreased. For grape skins, the O_2_^∙−^ content peaked on the second day of storage, and the overall accumulation content in the control group was 1.23 times higher than that in the treatment group. For grape pulp, the accumulated O_2_^∙−^ content in the control group and the treatment group reached a peak of 3.53 nmol g^−1^ and 2.19 nmol g^−1^ on the 8th day of storage, respectively, and was 37.9% higher than that of the treatment group. The overall H_2_O_2_ content of grape berry (Figure 4B) increased, and the H_2_O_2_ content in grape skins showed significant differences after the second day of storage. In grape pulp, the H_2_O_2_ content in the treatment group was always lower than that in the control group during storage, and there were significant changes except for the 2nd and 4th days (*p* < 0.05). On the 12th day of storage, the H_2_O_2_ content accumulated in the treatment group was 21.8% less than that in the control group. The MDA content of the skin and pulp is shown in Figure 4C: In the grape skin, the MDA content of the treatment group decreased slowly, while that of the control group increased first and then decreased, and the accumulated MDA content was 1.17 times that of the treatment group. On the 6th day of storage, the difference between the two groups was the most obvious, and the control group showed values 37.4% higher than the treatment group. In the pulp, the accumulation rate of MDA content in the treatment group was significantly lower than that in the control group (Figure 4C). In conclusion, CDs-mediated photodynamic treatment could alleviate the excessive accumulation of O_2_^∙−^ and H_2_O_2_, alleviate the oxidative damage of cell membranes and keep the reactive oxygen species level of grape berry in a low state, thereby delaying the onset of senescence and increasing the storage period. Du et al. [24] also reported that the use of CDs combined with photodynamic treatment of fresh fruit wolfberry could delay the accumulation of O_2_^∙−^, H_2_O_2_, and MDA. In addition, this was confirmed by Wang et al. [32] reporting the treatment of blueberries with methyl jasmonate.

### 3.5. Effect on the Content of Total Phenols, Flavonoids, and Reducing Sugars

**Figure 5:**
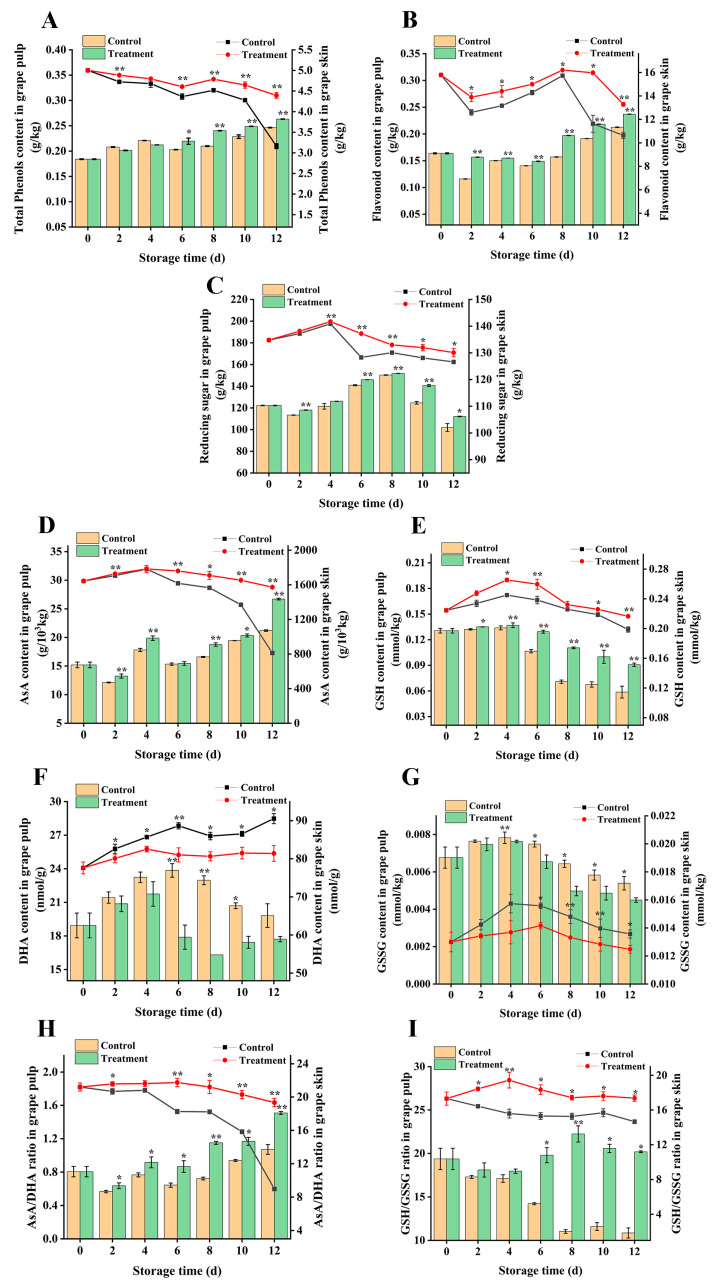
Effect of CDs-mediated PDT of total phenols content, flavonoid, reducing sugar, AsA content, GSH content, DHA content, GSSG content, AsA/DHA, and GSH/GSSG in grape (**A**–**I**). Treatment group: 0.06 g L^−1^ CDs solution and blue light irradiation for 5 min; control group: distilled water instead of treatment and storage at 25 °C for 12 days. * and ** represent significant differences between the sample point treatment and control groups, where * represents *p* < 0.05, and ** represents *p* < 0.01, and data are presented as mean ± SD (standard deviation) (n = 3).

Phenols and flavonoids are active antioxidant components of grapes, and their content reflects the antioxidant capacity of grapes. The total phenols and flavonoids in the grape skin were higher than those in the grape pulp during the whole storage period, which were 20 times and 82 times higher than the pulp (Figure 5A,B), indicating that the grape skin was rich in more phenols and flavonoids. Both grape skin and grape pulp accumulated more total phenol and flavonoid contents than control group and showed significant changes (*p* < 0.05).

In grape skins, the total phenolic content of both the treated and untreated groups decreased during storage, while the total phenolic content of the treated group was relatively stable, and the total phenolic content of the treatment group on the last day of storage was 1.38 times that of the control group. However, the total phenolic content in grape pulp showed a slight increase in the opposite trend of the skin, and the total phenolic content in the treatment group was significantly different at 6, 8, 10, and 12 days (*p* < 0.05).

After harvest, the flavonoid content of grape skin decreased firstly, then increased slightly from day 6, and then re-decreased from day 8; the treatment group maintained more flavonoid substances, and the flavonoid content at the end of the storage period was 28% which higher than that of the control group. The change in flavonoid content in grape pulp was consistent with that of total phenol, exhibiting a progressive increase. Overall, the treatment group showed a 1.12-fold higher total flavonoid content compared to the control group.

As a major energy substance, reducing sugar is a key source of energy for postharvest fruits. Throughout the storage period, the reducing sugar content of grape berry first increased and then decreased (Figure 5C), and the reducing sugar content in grape skin was higher during storage, which was 1.05 times higher than that of pulp. Compared to the control group, the reducing sugar content was consistently higher in the grape skin and pulp treatment group. Zhang et al. [33] treated table grapes with nitric oxide and suggested that nitric oxide could increase the total phenol and flavonoid content of grapes. In addition, it was found elsewhere that the treatment of grapes with spermine can improve their nutritional value and accumulate more phenolic substances, thereby delaying fruit senescence and rot [34], which is consistent with this study. Therefore, CDs combined with photodynamic treatment may also increase the content of phenols and flavonoids so that grape berries accumulate more natural antioxidants.

### 3.6. Effects on Antioxidants in the Ascorbic Acid–Glutathione Cycle in Grape Berry

To explore the antioxidant system of AsA-GSH to reveal the mechanism of reduced peroxidation in cell membranes, we performed experiments and found that CDs combined with photodynamic treatment inhibited the decrease in AsA and GSH contents (Figure 5D,E) and the increase in DHA and GSSG contents (Figure 5F,G) during storage. It is worth noting that the grape skin was rich in higher AsA content compared to the grape pulp. In the grape skin, the AsA content in the control group exhibited a rapid decline starting from the 4th day, ultimately decreasing by 51.3% on the 12th day of storage compared to the treatment group (*p* < 0.01). During the entire storage period, the treated group exhibited a 1.10-fold increase in AsA content in the pulp compared to the blank group. (Figure 5D). The DHA content in the skins and pulp of grapes increased first and then decreased throughout the storage period, peaking on the 6th day of storage, which was 1.09 and 1.33 times higher than that of the treatment group (Figure 5F). The same trend was observed in GSH and GSSG in grape skin and pulp, both of which showed an increase first and then a decrease. The GSH content of grape skin and pulp reached the peak on the 4th day (*p* < 0.01), which was 1.08 and 1.02 times that of the control group, respectively. (Figure 5E) The changes of GSSG in grape skin and pulp were the same as DHA, and the content of GSSG and DHA in treatment group was lower than that in control group (Figure 5G). The AsA/DHA ratio (Figure 5H) as well as the GSH/GSSG ratio (Figure 5I) in the treatment group exhibited significantly higher values compared to those in the control group, indicating significant differences (*p* < 0.05).

Fruit senescence is mainly due to damage caused by excessive ROS, and the non-enzymatic substances AsA and GSH in the AsA-GSH cycle can be used as ROS scavengers, and the involvement in cellular redox is of paramount importance [35], among which GSH is also an antioxidant, which can convert reduced dehydroascorbic acid into AsA again and continue to exert antioxidant effects [36]. In addition, the AsA/DHA and GSH/GSSG ratios reflect the redox state of the cell and also play an important role in the removal of ROS [37]. In present research, CDs combined with photodynamic treatment stimulated the accumulation of AsA and GSH in grape skin and pulp, reduced the content of DHA and GSSG, increased the ratio of AsA/DHA to GSH/GSSG, maintained a higher redox homeostasis than the control group, and improved the antioxidant capacity of grape berry. Fumigation of longan fruits with chlorine dioxide [38] and treatment of grapes [33] with nitric oxide and treatment of peaches [39] with methyl jasmonate improved the function of the AsA-GSH circulatory system, scavenging excess ROS and thereby delaying fruit senescence, which is line with the findings of this study.

### 3.7. Effects on the Activities of Antioxidant Enzymes in Grape Berry

**Figure 6:**
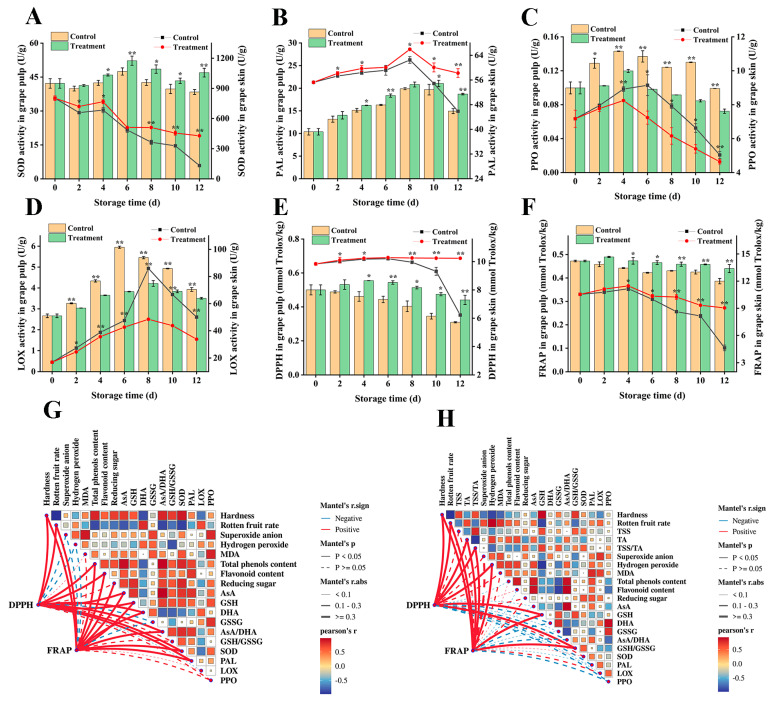
Effect of CDs-mediated PDT of SOD activity, PAL activity, PPO activity, LOX activity, DPPH, and FRAP in grape (**A**–**F**); carbon dots mediated the relationship between quality indexes, antioxidant indexes, and total antioxidant capacity indexes in grape skin (**G**) and pulp (**H**) in photodynamic treatment. The edge width corresponds to Mantel’s r-value, the color represents statistical significance, and the pairwise correlation of these variables is represented by the color gradient of the Pearson correlation coefficient. Treatment group: 0.06 g L ^− 1^ CDs solution and blue light irradiation for 5 min; control group: distilled water instead of treatment and storage at 25 °C for 12 days. * and ** represent significant differences between the sample point treatment and control groups, where * represents *p* < 0.05, and ** represents *p* < 0.01, and data are presented as mean ± SD (standard deviation) (n = 3).

With the extension of time after harvesting, ROS further accumulates, which can accelerate the deterioration of fruit quality after harvest, such as browning and rotting [40]. SOD is the first line of defense to remove ROS; PAL is a key enzyme in phenylpropanoid metabolism, which can improve its enzymatic activity and increase the resistance of phenols and flavonoids; and PPO and LOX reflect the degree of browning and damage of fruits. During the whole storage period, CDs combined with photodynamic treatment significantly increased the SOD activity and PAL activity and significantly inhibited the PPO activity and LOX activity of grape berry compared with the control group. The SOD activity in grape skin was observed to be 69% higher in the treatment group compared to the control group on the 12th day of storage. In grape pulp, SOD activity reached its peak on day 6 of storage and exhibited a 1.10-fold increase compared to the control group (Figure 6A). Overall, the treated grapes had higher SOD activity, which helped to scavenge ROS, which may have contributed to the reduction in superoxide anion and hydrogen peroxide. The PAL activity (Figure 6B) in grape skin exhibited higher levels compared to that in grape pulp, displaying a similar pattern of initial increase followed by decrease. Moreover, the enzyme activity in the treatment group consistently surpassed that of the control group. Compared with the control grape, the treated grape berry had lower PPO activity (Figure 6C) and LOX activity (Figure 6D) and showed a significant difference (*p* < 0.05). Ge et al. [41] demonstrated that treatment of blueberries with γ-aminobutyric acid could increase PAL enzyme activity and decrease PPO activity, which was the same as in this study. In addition, in this study, the PAL activity of grape berry increased later in storage, which may be the reason for the increase in total phenols and flavonoids, which was also confirmed by Zhang et al. [33] and Niazi et al. [42].

### 3.8. Effects on the Total Antioxidant Capacity in Grape Berry

The combined action of various antioxidant substances and enzymes in plants enhances the total antioxidant capacity of fruits, which can effectively remove excess reactive oxygen species molecules [43] and balance the life homeostasis of plant cells. In this study, CDs combined with photodynamic treatment effectively enhanced DPPH free radical scavenging capacity and maintained higher iron reduction antioxidant levels. On day 12, the photodynamically treated grape skin contained higher DPPH radical scavenging capacity (Figure 6E) and iron reduction antioxidant levels (Figure 6F), which were 39.25% and 48.83% higher than those untreated, respectively. Likewise, the total antioxidant capacity in the pulp of the photodynamically treated grapes was consistently higher than that in the untreated group. In our study, CDs combined with photodynamic treatment increased the content of total phenols, ascorbic acid, flavonoids, glutathione and other antioxidant substances in grape berry and enhanced the enzymatic activities of SOD and PAL, and the combined effect of these non-enzymes and enzymes enhanced the total antioxidant capacity of the fruits, which was in accordance with Zhang et al. [3] and Li et al. [44]. Our data signified that CDs combined with photodynamic treatment increased the total antioxidant capacity of grape berry, which reduced ROS damage, delayed fruit senescence after harvest, and prolonged fruit storage.

### 3.9. Correlation between Total Antioxidant Capacity and Quality Indexes and Antioxidant Indexes

According to the correlation network heat map, the correlation between the quality indexes and antioxidant line indexes and the total antioxidant capacity during grape storage is shown in Figure 6. In grape skin, fruit hardness was strongly positively correlated with total phenols, flavonoids, reducing sugars, AsA, GSH, AsA/DHA, GSH/GSSG, and SOD and negatively correlated with decay rate, DHA, and LOX. The degree of fruit decay was remarkably positively correlated with DHA and LOX and negatively correlated with total phenols, flavonoids, reducing sugars, AsA, GSH, AsA/DHA, GSH/GSSG, and SOD. It is worth noting that DPPH and FRAP, the total antioxidant capacity indexes, were strongly correlated with hardness, total phenols, flavonoids, AsA, GSH, AsA/DHA, SOD, and PAL and showed a strong positive correlation. In grape pulp, fruit hardness was strongly positively correlated with TSS, TSS/TA, GSH, and GSH/GSSG and negatively correlated with decay rate, O_2_^∙−^, H_2_O_2_, MDA, and LOX. The decay degree of fruit was strongly positively correlated with TA, O_2_^∙−^, H_2_O_2_, and MDA and negatively correlated with TSS, TSS/TA, GSH, and GSH/GSSG. In addition, DPPH and FRAP were strongly correlated with hardness, decay rate, TSS/TA, TA, GSH, and GSH/GSSG and showed a central positive correlation. (*p* < 0.05) (Advanced Cor link was performed using the OmicStudio tools at https://www.omicstudio.cn/tool; accessed on 10 September 2023). Overall, these correlations underscore the importance of antioxidants, enzymatic activities, and oxidative stress markers in determining the quality and preservation of grapes during storage.

## 4. Conclusions

As a photosensitizer-facilitating photodynamic technology, CDs could efficiently decrease the rate of Kyoho grape deterioration while in storage; preserve the grapes’ hardness, TSS, TA, and nutrients (reducing sugars); boost the accumulation of total phenols, flavonoids, AsA, GSH, and other antioxidants; prevent the accumulation of O_2_^∙−^, H_2_O_2_, and MDA; enhance the activities of SOD and PAL; and reduce the activities of PPO and LOX to lessen the oxidative damage of fruits. At the same time, it could also enhance the total antioxidant capacity of the fruit, thereby delaying the senescence of the fruit and prolonging the storage period. Therefore, CDs-mediated photodynamic technology has a good development prospect in fruit and vegetable preservation.

## Data Availability

The original contributions presented in the study are included in the article/Supplementary Material, further inquiries can be directed to the corresponding author.

## Supplementary Materials

The following supporting information can be downloaded at: https://www.mdpi.com/article/10.3390/foods13172717/s1, Figure S1: The real photo of the experiment using photodynamic processing equipment.
