# Supplementary material for: Carbon Dots-Mediated Photodynamic Treatment Reduces Postharvest Senescence and Decay of Grapes by Regulating the Antioxidant System
Source: Foods. 2024 Aug 27;13(17):2717. doi: 10.3390/foods13172717 (PMC11394370; doi:10.3390/foods13172717)
Supplement: Supplementary file 1 [file foods-13-02717-s001.zip › foods-3149962-supplementary.pdf]

Supplemental Fig. 1

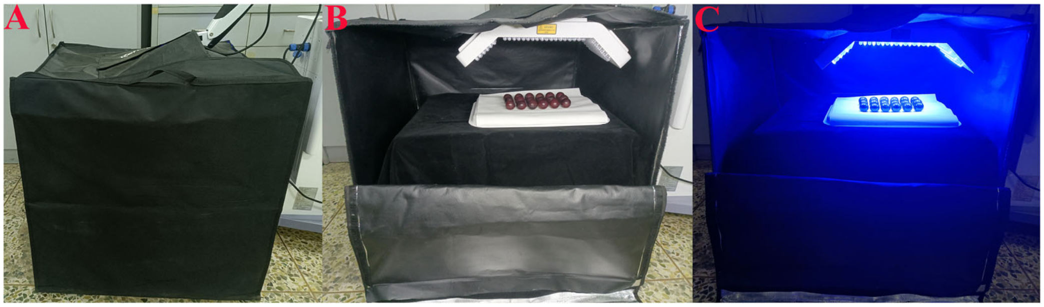

Supplemental Fig. 1. The real photo of the experiment using photodynamic processing equipment. A: The photodynamic processing equipment; B: The grape in photodynamic processing equipment; C: The grape under light exposure in photodynamic processing equipment. Actual distance from light source: 20 cm. The grape sample sprayed with 0.06 g/L CDs solution is placed on the work table without the sample rotating. Light source manufacturer: Wuhan Times Sunshine Technology Development Co., LTD.
